# Early intervention for children at risk of visual processing dysfunctions from 1 year of age: a randomized controlled trial protocol

**DOI:** 10.1186/s13063-019-3936-9

**Published:** 2020-01-08

**Authors:** Marlou J. G. Kooiker, Yoni van der Linden, Jenneke van Dijk, Ymie J. van der Zee, Renate M. C. Swarte, Liesbeth S. Smit, Sanny van der Steen-Kant, Sjoukje E. Loudon, Irwin K. M. Reiss, Kees Kuyper, Johan J. M. Pel, Johannes van der Steen

**Affiliations:** 1 Department of Neuroscience, Erasmus Medical Center, PO Box 2040, 3000 CA Rotterdam, The Netherlands; 2 Royal Dutch Visio, Center of Expertise for Blind and Partially Sighted People, the Hague, The Netherlands; 3 Royal Dutch Visio, Center of Expertise for Blind and Partially Sighted People, Rotterdam, The Netherlands; 4 Department of Pediatrics, Division of Neonatology, Erasmus MC-Sophia Children’s Hospital, Rotterdam, The Netherlands; 5 Department of Neurology, Division of Pediatric Neurology, Erasmus MC-Sophia Children’s Hospital, Rotterdam, The Netherlands; 6 Royal Dutch Visio, Center of Expertise for Blind and Partially Sighted People, Huizen, The Netherlands; 7 Department of Pediatric Ophthalmology, Erasmus MC-Sophia Children’s Hospital, Rotterdam, The Netherlands

**Keywords:** Visual intervention, Visual habilitation, Cerebral visual impairment, Neurological risk, Eye tracking, Visual attention, Visual processing, Brain damage, Children born preterm

## Abstract

**Background:**

An increasing number of children are suffering from brain damage-related visual processing dysfunctions (VPD). There is currently a lack of evidence-based intervention methods that can be used early in development. We developed a visual intervention protocol suitable from 1 year of age. The protocol is structured, comprehensive and individually adaptive, and is paired with quantitative outcome assessments. Our aim is to investigate the effectiveness of this first visual intervention program for young children with (a risk of) VPD.

**Methods:**

This is a single-blind, placebo-controlled trial that is embedded within standard clinical care. The study population consists of 100 children born very or extremely preterm (< 30 weeks) at 1 year of corrected age (CA), of whom 50% are expected to have VPD. First, children undergo a visual screening at 1 year CA. If they are classified as being at risk of VPD, they are referred to standard care, which involves an ophthalmic and visual function assessment and a (newly developed) visual intervention program. This program consists of a general protocol (standardized and similar for all children) and a supplement protocol (adapted to the specific needs of the child). Children are randomly allocated to an intervention group (starting upon inclusion at 1 year CA) or a control group (postponed: starting at 2 years CA). The control group will receive a placebo treatment. The effectiveness of early visual intervention will be examined with follow-up visual and neurocognitive assessments after 1 year (upon completion of the direct intervention) and after 2 years (upon completion of the postponed intervention).

**Discussion:**

Through this randomized controlled trial we will establish the effectiveness of a new and early visual intervention program. Combining a general and supplement protocol enables both structured comparisons between participants and groups, and custom habilitation that is tailored to a child’s specific needs. The design ensures that all included children will benefit from participation by advancing the age at which they start receiving an intervention. We expect results to be applicable to the overall population of children with (a risk of) VPD early in life.

**Trial registration:**

Netherlands Trial Register: NTR6952. Registered 19 January 2018.

## Background

Over the past decades, survival rates of children with neurodevelopmental disorders or who experienced adverse perinatal events (e.g., preterm birth, hypoxia/ischemia, cerebral hemorrhages) have increased due to the intensive, high quality care that they receive. As a consequence, the prevalence of children with brain damage has also increased. Since approximately 40% of the brain is involved in processing visual information, there is a high likelihood that these children will develop visual dysfunctions. Visual dysfunctions with a cerebral origin are generally called cerebral visual impairments (CVI) and have become common in children – conservative estimates range from 10 to 22 cases per 10,000 births in developed countries, to 40 per 10,000 births in developing countries [1]. CVI are now the primary cause of low vision in children [2, 3]. The visual system consists of an extensive network that involves both ocular and subcortical structures and numerous brain areas, including higher-order parietal and frontal areas. Consequently, children with brain damage-related visual impairments, or CVI, are a heterogeneous population that can suffer from a variety of problems – from lower-order visual sensory and oculomotor deficits, to higher-order visual perception difficulties. What they have in common on a functional level are challenges with detecting, attending to and processing incoming visual information, i.e., visual processing dysfunctions (VPD). VPD include issues with orienting and maintaining visual attention, detecting and perceiving specific visual features (e.g., colors, forms, motion) as well as with giving meaning to what is perceived and using the visual input for behavior. These visual processing functions can be considered prerequisites for higher-order visual perception functions, e.g., visual recognition and visuospatial orientation. Unlike such perception problems, which cannot reliably be assessed before school age, signs of VPD can be detected at earlier developmental stages. Given that early visual dysfunctions can severely delay or disrupt neurocognitive, motor and behavioral development [4–6], VPD particularly affect young children.

To alleviate early-onset problems, prevent later growing-into-deficit, and maximize developmental opportunities, detection and habilitation of VPD early in life is crucial [7, 8]. Early in development, high levels of cerebral plasticity may enable recovery or take-over of function on a structural level, whereas early and diverse visual experiences may enhance visual development on a functional level. Therefore, it is assumed that the sooner visual habilitation programs start, the higher the likelihood that they will stimulate and improve visual processing development. Although there are a large number of infant studies regarding early detection of visual dysfunctions that rely on this assumption, the effectiveness of early interventions in the visual domain has never been proven.

Early habilitation starts with early detection. Before the age of 4–5 years, it used to be challenging to assess the functional consequences of damage to the brain’s visual system. There have been advances in the early detection of (a risk of) visual problems, for example, the early assessment of basic visual function in neonates as early as 31 weeks of gestation [9] and a functional vision battery with cognitive and integrative aspects for use between 1 and 4 years of age [10]. These batteries involve various aspects of visual function and rely on behavioral observations. In addition, more quantitative, computer-based methods have been developed such as an eye tracking-based task for attention in toddlers [11]. Building on these innovations, our group developed a method to quantify visual processing efficiency in a non-verbal manner in children, using an eye tracking-based approach [12, 13]. We showed that children with (a high risk of) brain damage or dysfunction (e.g., children with visual disorders or children born extremely preterm) are prone to developing VPD [14–16]. These VPD were particularly strongly correlated with diagnosed CVI [17, 18]. These innovative early and non-verbal assessments can fulfill the need for scientifically strong psychometric tools to evaluate the effectiveness of early intervention. Besides providing an early characterization of VPD that was previously unavailable, they also open up the possibility to monitor or habilitate VPD at a young age. One can even argue that early detection of problems is only useful if it leads to advancing support and habilitation. Hence, the essential next step in the field of pediatric visual dysfunctions is to provide affected children with effective early intervention programs.

A major problem is that there is no evidence-based intervention for VPD from 1 year of age – this area is severely understudied [19] and quality of evidence is low [20–22]. Available (visual) interventions lack a standardized approach and/or systematic objective evaluation [23–25], and ideas about their effectiveness merely stem from clinical impressions, not from randomized and carefully controlled studies. The available evidence has predominantly been found in older children, for a limited range of visual functions (i.e., visual acuity and/or contrast sensitivity) [26], without incorporation of functional vision measures, using only stimulation and no training [24, 26], and without objective outcome measures. Studies that did use a comprehensive and structured training approach had a small sample size [27, 28]. Although these existing studies provide important information on the approaches and possibilities to habilitate visual problems, the effectiveness of visual intervention programs for children younger than 4 years has not yet been investigated with a randomized controlled trial (RCT).

VPD in children can arise from many different conditions, e.g., perinatal asphyxia or hypoxia, focal lesions, cranial trauma, infections, or hemorrhages. Therefore, the general population with VPD is a highly heterogeneous group of children. In the present study, we focus on children born very or extremely preterm (i.e., born < 30 weeks of gestation) from 1 year of corrected age (CA). In an ongoing longitudinal study, we found elevated risks of visual attention and processing problems in this population [15, 16], driving the urgency for interventions. However, we expect outcomes to be applicable to other young children at risk of brain damage-related VPD. After positive results have been obtained, the visual intervention program can be investigated in other risk groups.

The aim of this study is to investigate the effectiveness of early intervention of VPD in children born very or extremely preterm from 1 year CA. To this end, we developed a structured yet individually tailored visual intervention protocol. We will test its effectiveness in enhancing visual development with a RCT. Unique features of the presented approach are the objective outcome measures assessed from a young age, broad quantitative and qualitative data collection to assess the full spectrum of visual functions and neurodevelopment, and embedding of the project within standard neonatal and visual clinical care. This protocol was written in accordance with the Standard Protocol Items: Recommendations for Intervention Trials (SPIRIT) guidelines. Additional file 1 contains the SPIRIT checklist.

## Methods

### Study design

Randomized single-blind, placebo-controlled intervention study (RCT), embedded within standard clinical care.

### Study setting

The study will be executed at the Neonatology Department of an academic medical center, in collaboration with the Department of Pediatric Ophthalmology and with four regional centers of a center of expertise for blind and partially sighted people that provides visual diagnostics and (re)habilitation.

### Participant characteristics and timeline

It is expected that 25–50% of the very preterm population is at risk of VPD at 1 year CA [15, 16]. Therefore, all infants who have been born before 30 weeks gestational age and who participate in the clinical follow-up program of the Department of Neonatology, will be available for inclusion around 1 year CA. We aim to include children from 1 year CA, born < 30 weeks of gestation (about 50% are expected to be at risk of VPD, and eligible for the intervention) (*n* = 100) and children born at term without VPD from 1 year of age, to add to an existing database of typically developing children (healthy control group) (*n* = 100). The study population will be divided into three groups, with two subgroups – Group 1: children born very preterm from 1 year CA, without a risk of VPD; Group 2: children born very preterm from 1 year CA, with a risk of VPD (50% will receive direct visual intervention (group 2A, intervention group) and 50% will receive postponed visual intervention (group 2B, control group); Group 3: children born at term without a risk of VPD.

### Eligibility criteria

Inclusion criteria

- Born < 30 weeks gestational age
- Age at inclusion of 1 year CA (+/− 2 months)

Exclusion criteria

- Visual acuity below 0.05 (Snellen equivalent); the visual screening (i.e., eye tracking-based exam) is designed to be visible with a visual acuity of 0.05 or higher
- High chance of epileptic activity during assessment; more than two attacks in the previous year or when using the anti-epileptic vigabatrin (which may lead to visual dysfunction)
- Retinopathy of prematurity of grade 3 or higher, assessed by a pediatric ophthalmologist, as this will account for their visual dysfunction

### Study procedures

**Fig. 1:**
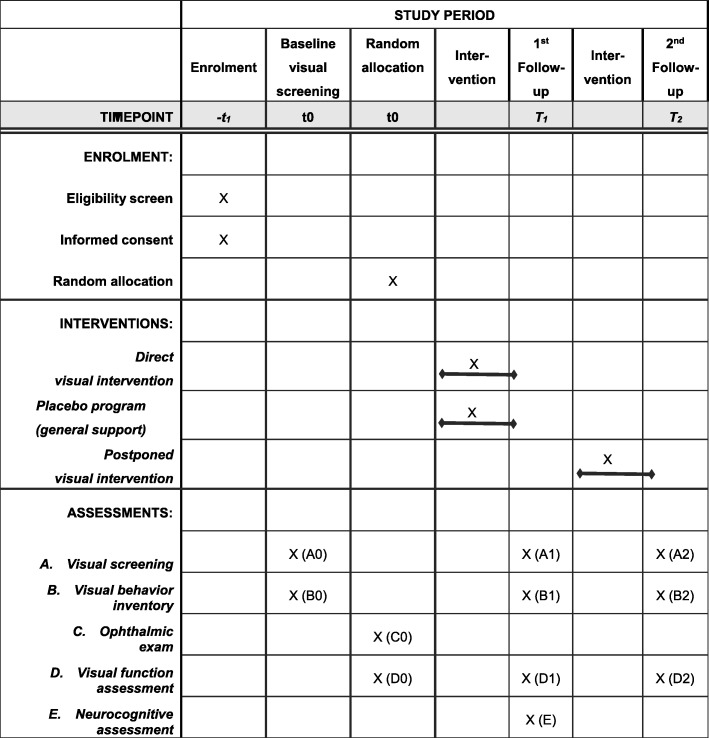
Time schedule of enrolment, interventions, and assessments

**Fig. 2:**
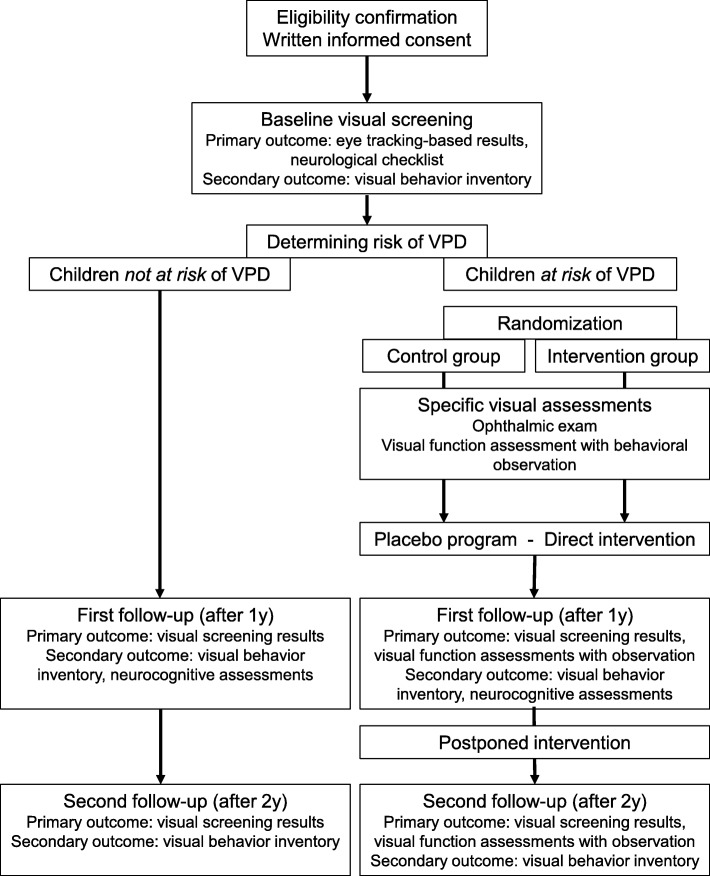
Flow diagram

Figure 1 outlines the general time schedule of the study. Figure 2 outlines the flow of assessments and interventions for study participants.

#### Participant inclusion and baseline screening

Children born at < 30 weeks gestational age will be recruited around the CA of 1 year at the Department of Neonatology of an academic medical center. After obtaining written informed consent (Additional file 2), a visual screening is performed to identify the prevalence and nature of VPD (for details, see ‘Data collection methods’). The first part of the screening consists of an eye tracking-based assessment of visual attention and processing. The visual assessment results are compared to normative references to identify the children with a risk of VPD. The second part of the screening is a checklist to identify common neurological risk factors for VPD, and that is completed by the neonatologists.

Children who are identified as being at risk of VPD will first undergo an orthoptic and ophthalmic exam at the Department Pediatric Ophthalmology (including refraction, ocular alignment). Next, they will be referred to a visual advisory and rehabilitation center in order to receive standard care, consisting of a visual function assessment (VFA) and a visual intervention program. The VFA is used to evaluate visual sensory functions (e.g., visual acuity, visual field, contrast sensitivity, ocular motility) and to observe the functional visual behavior of the child. This assessment is performed by experienced orthoptists, optometrists and behavioral therapists, and together they will determine the visual level of the child [5, 29] according to the following categories:

A. Profound visual dysfunction/legally blind (mainly responding to light)
B. Severe visual dysfunction, passive attentional system (reactions to stimuli do not reach normative levels, child does not actively search for visual stimulation, low recognition)
C. Moderate visual dysfunction/basic perception (active visual attention system and basic visual recognition)
D. Mild visual dysfunction, subnormal visual function (functioning at the lower bound of normal)

#### Intervention

For a detailed outline of the visual rehabilitation protocol we refer to the section ‘Intervention: a visual habilitation protocol’ and to Additional file 3: template visual intervention protocol. To reliably examine the effectiveness of the visual intervention program with an RCT, children who are at risk of VPD will be randomly allocated to one of two groups, as follows:

1. *Intervention group (direct)*

This group consists of very preterm children who are at risk of VPD and who will start the visual intervention program upon referral to the visual rehabilitation center (i.e., around 1 year CA). The program consists of a general protocol (standardized across participants) and a supplement protocol (tailored to the child’s specific VPD), and lasts ~ 1 year.

2. *Control group (postponed and placebo)*

This group consists of very preterm children who are also at risk of VPD, but for whom the visual intervention program will be postponed for the duration of 1 year. These children will be placed on a waiting list for the duration of the direct visual intervention (i.e., ~ 1 year). During this first year, they will receive a placebo intervention involving general developmental support that is aimed at monitoring the child’s developmental progress without providing specific visual habilitation. As soon as the follow-up assessments of the direct intervention group are completed, children in the control group will start visual intervention (i.e., around 2 years CA).

Importantly, this study design ensures that all children at risk of VPD will receive visual intervention at an earlier age than is the case in current standard care (i.e., where only a small number of young children will be referred based on obvious ocular disorders, and others will not receive interventions before the age of 4–6 years), while the RCT design enables a reliable and controlled comparison of the effectiveness of visual intervention within this group.

#### Follow-up after 1 year

One year after inclusion, the children at risk of VPD will repeat the visual function assessments, and all included children (with and without VPD risk) will repeat the eye tracking-based visual screening and undergo a neurodevelopmental assessment (that is standard care at most Neonatology departments, from 2 years CA). That way, the specific effects of early visual intervention on visual processing and neurocognitive development are compared and evaluated.

#### Postponed intervention

After the first follow-up, the children in the postponed intervention group will start their visual intervention program, which will also be evaluated 1 year later. In addition, the results of the visual screening are again evaluated for all children, to identify new cases with a risk of VPD who will then also qualify to start visual intervention. Differences in effectiveness of direct and postponed early visual intervention are assessed. The intervention study will have a duration of either 1 or 2 years, depending on the visual intervention group.

### Data collection methods

#### Baseline (T0)

Medical and demographic information will be extracted from the medical records available at the academic medical center.

##### Visual screening – A0

All included children will be screened for a risk of VPD, which consists of (1) an eye tracking-based assessment and (2) a neurological checklist. The eye tracking-based assessment is used to measure visual attention and processing functions. The assessment will be combined with an existing appointment for standard outpatient visits at the Neonatology Department when the child is ~ 1 year CA (T0) and 2 years CA (T1). During this assessment, children sit in front of the eye tracker monitor at a distance of approximately 60 cm, either independently, on the lap of their parent or in a pram. They do not receive verbal instructions and their body and head position is not restricted. The assessments are conducted in a quiet room with ambient light conditions. Visual stimuli (images and movies) are presented on the monitor to engage the reflexive orienting eye movements of the child, while simultaneously the eye positions are recorded over time using infrared cornea reflection (Tobii T60 XL or Tobii X3, Tobii Corporation, Danderyd, Sweden). That way, the child’s eye movement responses to various types of visual information (i.e., contrast, color, motion and form) are automatically recorded. These viewing behavior responses indicate whether and how fast a specific stimulus was detected and looked at. From these responses, the number of detected stimuli and the reaction time to fixation (RTF) of a stimulus are calculated. RTF is a measure for the timing of detecting and processing visual information and is the main study parameter [12, 13, 30, 31]. Total test duration is approximately 15 min. The child’s viewing behavior parameters per visual stimulus are analyzed and compared with normative data, i.e., developmental trajectories of an existing database of healthy control children, born at term to identify abnormal detection and timing of processing of the visual stimuli. This results in a classification of normal and abnormal visual attention and processing functions.

Second, medical specialists examine the child’s medical history for the presence of neurological risk factors for VPD in the context of prematurity [32–35], i.e., moderate-severe damage on neonatal MRI scans; cerebral palsy, unilateral/bilateral hemiplegia/diplegia; infantile esotropia/convergent strabismus or nystagmus; and deviating head circumference (> 1 SD in 12 months).

##### Inventories for daily life visual functioning – B0

The Participation and Activities Inventory – Children and Youth (PAI-CY) 0–2 questionnaire [36] will be filled in by parents upon inclusion. This questionnaire assesses daily visual functioning in seven domains. Originally developed as a list to identify participation and activity needs, it can also be used to investigate and monitor intervention needs of visually impaired young children. It is the only available patient-reported visual outcome measure for young children and has satisfactory psychometric properties [37].

##### Determining the risk of VPD

The risk of VPD is determined based on the baseline screening: abnormal viewing behavior, indicated by abnormal RTF values on one or more visual stimuli and/or the presence of at least one neurological risk factor for VPD. The children at risk of VPD are referred to standard care for children with suspected visual dysfunctions, i.e., they will undergo an ophthalmic exam to evaluate eye and orthoptic function and they will become clients of the visual advisory and rehabilitation center where they undergo a visual function assessment and are enrolled in the visual intervention program.

##### Ophthalmic exam (standard care; group 2A and 2B) – C0

All children at risk of VPD will be referred to the Pediatric Ophthalmology Department to evaluate visual acuity, refractive error and ocular alignment. This evaluation is performed by ophthalmologists and/or research orthoptists. The total duration of the exam is approximately 1 hour.

##### VFA, (standard care, groups 2A and 2B) – D0

All children at risk of VPD will undergo an extensive VFA. This assessment is part of standard care and will be done by an experienced orthoptist or optometrist. All assessments will be performed according to a standardized protocol that ensures similar assessments, choice of tests and scoring by the various examiners. The following functions will be assessed: ocular alignment and fixation preference, binocular vision, presence of nystagmus, oculomotor function (fixation, saccades, pursuit, motility), convergence, visual acuity, visual field, contrast sensitivity and color vision. Performance per function is classified as normal or abnormal for the child’s age.

#### First follow-up (T1)

Starting at 1 year after inclusion, i.e., from 2 years CA, the following assessments are repeated:

##### Visual screening (study-specific; all groups) – A1

The visual assessment (eye tracking-based exams) will be repeated for all included children and will be combined with an existing appointment for standard outpatient visits at the Neonatology Department.

##### Inventory for daily life visual functioning (study specific) – B1

Parents are asked to complete the inventory again around the time of first follow-up.

##### VFA (standard care) – D1

The VFAs will be repeated in the children at risk of VPD (independent of the visual intervention group they are in), as part of standard care at the visual advisory center.

##### Neurodevelopmental assessment (standard care) – E

From 2 years CA, all children will receive a neurodevelopmental assessment at the NICU as part of the standard follow-up program of the Department of Neonatology. This assessment consists of the Bayley Scales of Infant and Toddler Development (Bayley-III-NL) and is performed by experienced (neuro)psychologists.

#### Second follow-up (T2)

Two years after inclusion, i.e., from 3 years CA, all included children will repeat the eye tracking-based visual screening (A2) and the inventories for daily life visual functioning (B2). Since at 3 years CA there is no regular follow-up within clinical care, study-specific appointments will be made at the academic hospital or in the form of home visits. In addition, the children at risk of VPD who have been referred to the visual advisory center will undergo the VFA again (D2) as part of standard care.

By embedding the majority of this study within standard clinical care, by closely collaborating with involved medical and visual (re)habilitation specialists, and by planning participation together with regular appointments or in the form of home visits, we expect to maximize participants’ completion of the follow-up measurements.

### Intervention: visual habilitation protocol (Additional file 3)

We developed a structured visual intervention protocol using a two-step approach – (1) dissecting available scientific knowledge about visual interventions in young children and (2) establishing other, clinically relevant, factors in close collaboration with experienced behavioral therapists and neuropsychologists.

First, based on the available (visual) intervention literature, we extracted several key features for the intervention, thus constructing a protocol with the following characteristics:

1. Starts well before school age to maximize experience-dependent neuroplasticity [38, 39]
2. Involves the total spectrum of visual development, not restricted to only a few visual functions [40] or general neurodevelopment [41]
3. Has quantitative and functional outcomes [23, 28]
4. Employs two intervention strategies that have complementary value [20, 27]:
  - Passive (bottom-up-feedforward) visual stimulation that is purposeful and specific [42–44]
  - Active (top-down modulated) visual perceptual training that is contingent on children’s abilities [28, 43, 45]
5. Can be individually tailored by adapting materials and activities to children’s preferences and capabilities [25]
6. Incorporates children’s systems through active caregiver involvement [38, 46]

Second, we examined the clinical and practical requirements for an intervention protocol by consulting professionals about which types of habilitation to include, in which (developmental) domains, which elements, materials and objects to use, minimum duration and frequency, and how to handle parental motivational and resistance issues. The answers to these questions were grouped and analyzed in order to extract common themes and select the most important clinical features the protocol should contain.

The result of this two-step process is a visual intervention program that consists of:

1. A general protocol that is identical for all children
2. A supplement protocol that is tailored to the specific VPD of the child.

Both parts are designed to adhere to the child’s basic visual skills and to their cognitive, motor and socioemotional developmental level. Importantly, the parent–child relationship will be taken into account in order to support, involve and stimulate the parents in executing the intervention program at home.

The general visual protocol is adapted to the child’s age and developmental level and consists of exercises focused on the following functions: fixation, pursuit, visual attention, enhancing visual experiences and knowledge, perception of details and combining vision with motor action (visuomotor skills). The intervention program consists of several steps that can be applied to all functions:

- Enhancing the diversity of training with different visual materials
- Enhancing the duration of visual training
- Developing increasingly complex visual skills and behavior that are ecologically valid, i.e., related to the child’s activities and daily environment [20].

Visual input is provided in the form of different visual materials of different visual modalities (colors, black-white, moving and static objects, light and dark). The nature of visual input will be adapted to the preferences and abilities of the child, and successful responses and behavior will be rewarded (based on operant conditioning). Visual training of more complex skills and behavior is done by teaching the child to use a specific visual skill, to expand its use to other tasks and to integrate it in everyday life.

The supplement visual protocol is designed around the specific VPD that are determined based on results of the visual screening and assessments (VFA and observation) performed at baseline. For example, children with abnormal processing of form and motion information, but with normal processing of contrast and color information, will get additional training for the processing of form and motion-related visual information that is integrated within the visual intervention program itself in order to comprehensively support the child.

Additional components:

- Focus moments. These therapy sessions with video feedback, evaluation with the parents and reports of behavioral observations, are done three times throughout the intervention period to determine whether the program suffices or needs adaptations.
- Stepping cards (per therapy session). An instruction for the daily practice sessions of parents that contains a specific goal, instruction, observational points, and evaluation.
- Logbook for therapists (per session) and parents (to be completed weekly). The logbooks are used to keep track of the frequency, intensity and content of the therapy sessions and the daily practice sessions by parents.
- Protocol for Activities & Materials to be used, based on chapter 5 [29]

### Criteria for discontinuing or modifying interventions

Modifying the intensity of intervention: after the second focus moment (around week 16), it will be determined whether the child will still benefit from weekly sessions or could have these every 2 weeks. If this intervention intensity is no longer needed, the intensity is brought down to once every 4 or 6 weeks, to keep monitoring (visual) development, and to enable another frequency adaptation when needed. This will be done until the end of the program.

Modifying the content or focus of rehabilitation: this is based on the evaluations with parents and the observations after each therapy session. If a modification is warranted, only the supplement protocol will be modified, the standard protocol will not change.

### Improving and monitoring intervention adherence

All intervention activities are demonstrated and explained to the parents by the therapists. The goal is to have them understand the content of activities and the underlying motivation, to stimulate parents to practice daily with their child. Parents will participate in all therapy sessions – they do not only receive practice instructions but will also be educated on the visual development of their child. The parents are asked to log their daily practice session in order to evaluate them in the therapy sessions. These evaluations will give insights in improvements and/or changes in visual performance of the child, which is known to motivate parents and enhance intervention adherence.

### Permitted concomitant care

Participating children will not be restricted in receiving care as usual. If applicable, they are allowed to engage in additional neurodevelopmental training programs during this study. However, participation in such programs will be carefully registered and monitored to consider interference with the possible effects of the visual intervention program. In addition, children in the control intervention group will be provided with general developmental support and monitoring that does not include specific visual training components. This program consists of visits by a psychologist-in-training (under supervision of neuropsychologists from the academic hospital and visual advisory center) and aims at monitoring developmental achievements of preterm children from 1 to 2 years of CA. That way, we directly involve all parents in the study, and enable a structured and controlled investigation of the effectiveness of specific visual habilitation. After concluding the study, the children who are at risk of VPD and have been referred to the visual rehabilitation center will continue to be monitored there. This means that their treatment does not necessarily stop, but that this depends on the indications and judgement of the therapists and psychologists.

### Outcome measures

To answer our main research question about the effectiveness of early visual intervention in young preterm children, we will compare visual outcomes between the intervention and control group. We will analyze results from the eye tracking-based visual assessment and the additional VFAs at baseline and at yearly follow-up measurements. Primary outcomes are the changes in visual parameters after the duration of the intervention study (i.e., from baseline to follow-up T1 and/or T2), namely quantitative visual parameters (eye tracking-based visual detection and viewing reaction times) and VFA (visual acuity, contrast sensitivity, visual field, ocular motility).

Endpoints are the changes in these parameters after the visual intervention program. These parameters are chosen since their combination provides quantitative and objective results about functional viewing behavior (the eye tracking-based assessment) and clinically relevant visual function outcomes (i.e., the VFAs). In addition, we will compare the effectiveness of direct (at 1 year CA) versus postponed visual habilitation (at 2 years CA) by comparing visual outcomes between these groups at T2.

Secondary outcomes are the neurodevelopmental level at the first follow-up (T1) and results from parental questionnaires about daily visual functioning at T1 and/or T2, i.e., total score, cognitive and motor sub scores of the neurocognitive assessment with Bayley Scales of Infant and Toddler Development (Bayley-III-NL) and total score and sub scores per visual domain of the PAI-CY 0–2 Inventory (i.e., attachment, stimulus processing, orientation, play, mobility, communication). These measures were chosen because Bayley-III-NL is the only available neurocognitive assessment for children at this age, and the PAI-CY 0–2 Inventory is the only available parental questionnaire about daily (visual) behavior at this age.

### Sample size calculation

Each year, approximately 200 children are born before 30 weeks of gestation and admitted to the Neonatal Intensive Care Unit at the academic medical center in Rotterdam, the Netherlands. Based on previous research with this population we expect an inclusion rate of ~ 50%. Therefore, we expect to include at least 100 children at 1 year CA. There are no thorough estimates of the prevalence of VPD in preterm children. A previous study using the eye tracking-based assessment in extremely preterm children identified visual processing delays in 48% of preterm children without evidence for brain damage [16], and in 9% to 23% of children in a cross section of the preterm population at 1 year CA [15]. In addition, between 25% and 33% of children with CVI have prematurity as a contributing factor [32]. However, the prevalence of risk factors for VPD in our study population (often with brain damage and perinatal events) is much higher than 50%. Therefore, we expect that each year at least 50 newborn preterm children from the Neonatology Department (i.e., 50%) are at risk of VPD and thus eligible for inclusion in the intervention study.

### Recruitment strategies

Children will be recruited from a medical follow-up program for children born preterm that is ongoing at the Department of Neonatology. Children’s eligibility for inclusion will be screened by a multidisciplinary team (project leader and project members from Neonatology). The project leader and/or project members will approach their parents first by telephone, to ask for permission to send an information leaflet about the study. Two weeks after sending the study information, parents will be contacted again to ask for their permission to include their child in the study. The baseline assessment (i.e., the visual screening) and the 1-year follow-up assessment will be scheduled together with an existing appointment at Neonatology, to minimize burden for children and parents. Children in the control group (group 3) will be recruited through daycare centers. The parents will receive study information by mail and are asked to contact the project leader if they are willing to participate. If they give their consent, an appointment for the visual assessment will be made.

### Randomization and intervention allocation

Prior to recruitment of participants, a randomization scheme has been designed in which the order of visual intervention groups (either direct or postponed) is randomized using an online tool (Sealed Envelope Ltd. 2016; https://www.sealedenvelope.com/simple-randomiser/v1/lists).

Randomization is blocked to ensure a balance in sample size across groups over time and stratified to control and balance the influence of covariates [47]. Covariates in the current study are the presence of brain damage and gestational age (< 28 weeks or 28–30 weeks).

After this computer-generated randomization, preterm children who are classified as being at risk of VPD will be assigned a participant number and concurrently be allocated to one of the visual intervention programs. The order of allocation corresponds with the date and order of inclusion. This allocation will be done by the principal investigator and remains concealed to all other investigators, the participating children, and their parents. All participant numbers will be separately placed in opaque envelopes that contain a note with the assigned visual intervention group. Revealing the group allocation to parents will be done by opening the opaque envelopes in their presence.

### Blinding

The study set-up is single-blind. Intervention allocation will be known by the project leader managing the contacts between all involved parties, and by the behavioral therapists of the visual rehabilitation center who will perform the interventions. Parents cannot be blinded to intervention allocation either, as they will actively participate in the intervention programs. However, the allocation will be blinded for the researchers and orthoptists who perform the baseline and follow-up visual assessments, and for the researchers performing data analyses.

Prior to analyzing results of the visual intervention (i.e., analyzing results of the follow-up visual assessments), participant numbers will be converted into a new participant code and visual intervention group will be coded into a new variable (group A or B). That way, group allocation remains concealed and bias during data analysis will be prevented [48].

### Data management

All data will be handled confidentially and anonymously. Communication about participating children between the various participating departments and organizations will only be done using codes, not names or other personal identifying information. All data will be coded, and a separate coding list will link study data to personal identifying information of a specific subject. The coding list is password-protected and only accessible by the main investigator(s). Data files will be stored on a PC and will be accessible with a password that is only known to the main investigator(s). Paper documents will be stored in a locked cupboard of which only the main investigator(s) will have a key. The investigator(s) will remain blind to the participants’ outcomes during the course of the study. All data will be anonymized prior to analyses and publications. Group allocation and randomization will not be revealed until after the statistical analyses have been finished.

### Withdrawal

Parents and their children can withdraw from the study at any time for any reason if they wish to do so without any consequences. The investigator can decide to withdraw a subject from the study for urgent medical reasons. When subjects withdraw from the visual intervention program they will not be replaced in order to ensure adherence to the randomization protocol for the RCT. Withdrawal of subjects will be taken into account during data analyses by using intention-to-treat analyses. Subjects withdrawn from the visual intervention program will be followed-up with a short questionnaire (assessed by telephone) in which they are asked for their reasons and circumstances of their withdrawal.

### Statistical analyses

#### Sample size calculation

To answer our primary research question about the effectiveness of early visual intervention programs on visual attention and processing functions in preterm children at risk of VPD, we use a repeated measures analysis of variance (ANOVA) for the parameter RTF as primary analysis. An a priori power analysis provided a recommended total sample size of 32 children (and an actual power of 0.81 and a critical F of 4.17).

#### Effectiveness of visual intervention program

In children at risk of VPD who have been allocated to a visual intervention group, differences in viewing behavior parameter RTF between T0 and T1 are analyzed with a Repeated Measures ANOVA. A *p* value of 0.05 will be considered statistically significant. Covariates are the medical (perinatal and ophthalmic) and demographic factors and the repeated measures analyses will be done with and without them to obtain their contribution to the main effect. All analyses are done according to the intention-to-treat principle.

#### Other effects of visual intervention on VPD (other parameters)

Secondary study parameters are the differences in other eye tracking-based parameters of viewing behavior and the outcomes of VFAs (e.g., visual acuity, contrast sensitivity, extent of visual field). Repeated measures ANOVAs with a *P* value of 0.05 will be considered statistically significant. After finding a main effect of group on the viewing behavior parameters, post-hoc comparisons will be done to establish which of the five visual stimuli significantly differ from each other in parameter values. For these comparisons, Bonferroni’s correction will be applied to the viewing behavior variables to correct the *P* value for the number of comparisons.

#### Effects of visual intervention on neurodevelopment

At 2 years of age, parameters from Bayley-III-NL are used as indicators of neurocognitive development. First, the relation between the visual assessment parameters and Bayley-III-NL parameters of the cognitive composite score, the language composite score, and the motor composite score will be analyzed using Pearson and Spearman correlation analyses. Next, univariate ANOVA’s are used to analyze differences in Bayley-III-NL scores between preterm children without a risk of VPD and children at risk of VPD, and between preterm children at risk of VPD in the direct versus the postponed intervention group.

### Monitoring

#### Data monitoring and auditing

The risks of our study for patients and for scientific quality have both been judged as negligible, based on the NFU risk classification (Netherlands Federation of University Medical Centers). This risk level implies that monitoring should be done once a year. Monitoring involves:

- Study documents and agreements
- Patient inclusion, consent, compliance, and source document verification
- Patient safety
- Study procedures
- Clinical data management system
- Correct saving of raw data, corrected data, and backups

#### Harms

In accordance with section 10, subsection 4 of the Wet Medisch-wetenschappelijk Onderzoek met mensen (WMO), the sponsor will suspend the study if there is sufficient ground that continuation of the study will jeopardize subject health or safety. The sponsor will notify the accredited Medical Ethical Testing Committee (METC) without undue delay of a temporary halt including the reason for such an action. The study will be suspended pending a further positive decision by the accredited METC. The investigator will take care that all subjects are kept informed.

### Ethics and dissemination

The present study has been approved by the METC of Erasmus Medical Center, Rotterdam (MEC-2016-724), on April 19, 2017. Important protocol modifications have been and will be communicated with the METC (in the form of amendments to the original protocol), project employees/researchers, and participating parents. Written informed consent will be obtained by the researchers before the baseline assessment, based on a comprehensive information document that the potential participants have received. See Additional file 2 for a model consent form.

Participant confidentiality will be protected through our data management policy (see section Data Management). The main investigator(s) and the participating medical and clinical specialists will have access to the final trial dataset. There are no contractual agreements that limit such access for investigators. During the course of the study, the principal investigator will have no access to patient-identifying data and communication will only be done using participant codes. The involved researchers and sponsors have no financial or other competing interests for the overall trial nor for each study site. No harm is expected from trial participation, in particular since most study components are part of standard clinical care. Therefore, ancillary and post-trial care are suspected to be non-applicable.

### Nature and extent of the burden, risks and benefits associated with participation

The risks associated with participation are negligible and the burden for the children is minimal. Apart from the visual assessment from 1 year CA onwards, the total program is standard care for children with (suspected) visual problems. Only the age at which this care is applied is advanced for this study, and the visual rehabilitation protocol has been structured in order to enable comparisons between children. There is a general burden for children to perform the study-specific assessments and for parents to accompany their children. A specific burden for parents of children in the intervention groups is the time and effort they are asked to invest in monitoring and logging the child’s daily practices in the home environment. Benefits for all preterm children are earlier visual assessments, general developmental support and, if applicable, earlier habilitation of a risk of VPD than is the case in conventional pediatric care (i.e., from 1 to 2 years CA instead of ~ 4 years CA).

### Dissemination policy

Dissemination of the results will include publications in peer-reviewed scientific journals and use of the visual assessments and the intervention protocol in clinical practice. There are no restrictions in the publication policy. The investigators aim to publish all results obtained from the study unreservedly.

### Authorship guidelines

Our study adheres to the Research Code of Erasmus MC in which guidelines for publishing and authorships are defined (https://www6.erasmusmc.nl/cs-research/bijlagen/publiceren?reason=404).

### Plans for granting public access to full protocol, dataset and statistical code

Public access to the full protocol will be given through this paper. All results will be published open access. The dataset will not be made publicly available given the patient identifying information it contains. Statistical code will be made available upon request.

## Discussion

The aim of this RCT is to investigate the effectiveness of an innovative and comprehensive visual intervention program for children with (a risk of) VPD from 1 year of age. We have developed a visual intervention protocol that adheres to recent scientific insights regarding habilitation and that exploits clinical expertise from visual rehabilitation centers in the Netherlands.

The present study provides a solution to some notorious problems in the (early) visual intervention domain. Firstly, an underlying problematic paradox in the execution of intervention studies is that the content of visual habilitation has to be tailored to the individual child in order to exert effects, but that establishing scientific evidence for effectiveness requires structured and controlled designs with homogeneous groups. With this protocol, we can achieve visual habilitation that is not only structured and evidence based, but also individually tailored and adaptive to a child’s level of functioning. Parts of the general visual protocol are currently used in visual practice but they have never been structured into one comprehensive protocol, and have never been the subject of scientific investigation. Since there is a lack of evidence-based visual intervention or training programs [20, 22], but clinical experiences are positive, these programs are considered best clinical practice. In addition, the supplement visual protocol and additional components were specifically designed for this study and satisfy the need for individually adaptive programs. Secondly, in order to obtain true evidence for the effects (does improvement occur as a result of the intervention or as result of ‘normal’ visual development?) an RCT is needed in which children with visual dysfunctions are randomly allocated to an intervention or control group. However, a difficulty with conducting RCTs in (young) patient groups is the ethical consideration regarding randomization of treatment allocation, which means that one group is withheld treatment. Our RCT design circumvents this problem. We start with providing intervention to all participants a couple of years earlier than is currently feasible within clinical visual care (from about 4 years of age). That way, both the intervention (from 1 year of age) and control group (from 2 years of age) will receive earlier care than usual and therefore are both expected to benefit from participation in this study.

This study has the potential to satisfy a great clinical and scientific need for early and evidence-based visual intervention options. To date, several reviews of visual interventions in children mentioned a lack of evidence-based programs. One showed some evidence for visual training as opposed to, more passive, visual stimulation [20]. Another focused on several strategies for visual improvement in children with frequently co-occurring visual and neurodevelopmental problems [22]. The strongest evidence was found for visual aids (e.g., spectacles) and environmental modification to compensate for visual loss. Less evidence was found for functional behavioral methods that focused on actually improving visual function, and it was stressed that more information on this subject is needed. Given the increasing prevalence of children with corrected low vision, i.e., for whom spectacles cannot provide more functional visual improvement, the availability of behavioral training programs is of the utmost importance.

It is important to note that the present study not only concerns early habilitation but also early screening of a VPD risk. A combined approach of detecting and habilitating VPD in children will benefit from the multidisciplinary collaboration of involved neuroscientists, neuropsychologists, and behavioral therapists. Upon achieving the study goals, this set-up also ensures immediate dissemination in the form of clinical implementation of the visual intervention program. The proposed training programs contain a unique combination of elements that ensures incorporation of a widely advocated system approach [38, 46]. This may not only improve VPD and their development, but also support the parent–infant relationship and improve infant self-regulation and later independence. Through the implementation of this new program, we expect that more children will gain opportunities for learning, development, and daily independence earlier in life. These are invaluable and essential steps toward an inclusive society that maximizes children’s opportunities.

The broader scope of this new intervention program lies in monitoring and supporting the development of children at risk, not only in the visual domain but also in behavioral, cognitive, and social-emotional domains. Focusing our attention on the early development of children born preterm will help set proper circumstances for further learning and development up to school age. Ultimately, we expect the outcomes to be applicable in all types of pediatric patients at risk of VPD (e.g., comorbid with syndromes or developmental disorders). The possibility to train visual functioning brings us closer to enhancing neurodevelopment in prevalent neurological risk groups and will optimize recovery or compensation on a functional and daily level.

### Study design challenges and limitations

An important challenge is to determine the right age to start interventions – how early is too early? This is yet unknown, which is why we chose to start at 1 year CA when basic visual and neurological development has completed and more developmental processes emerge. We expect that careful monitoring of visual assessment outcomes, not only related to interventions but also in the placebo control group and in the form of yearly follow-up measurements, will reveal the age(s) at which children start to show specific VPD. This will inform future clinical applications or new studies about the ideal developmental stages to start visual intervention programs. In addition, it was difficult to estimate the risk of VPD in this specific population of children born < 30 weeks at 1 year CA. The precise number will determine the inclusion and sample size of the intervention leg of the study. However, besides investigating effectiveness of visual intervention in children born preterm, the present protocol provides a solid basis for other studies and/or applications in different pediatric populations at risk of VPD early in life. Lastly, the study population is a relatively vulnerable risk group, particularly because of their young age. Children born very or extremely preterm generally go through a rough first start in the neonatal period, putting a medical and psychological burden on both the children and their parents. Therefore, we start recruiting at 1 year of CA when, for the majority of children, the most intense medical issues are behind them and caregivers may be open to exploring new developmental possibilities.

## Trial status

Protocol version number and date: 5, 19 April 2017.

Date recruitment began: 20 April 2017.

Date recruitment for visual screening completed: 1 June 2018.

Date recruitment for (direct and postponed) rehabilitation is estimated to be completed: 1 September 2019.

## Supplementary information

**Additional file 1.** SPIRIT 2013 Checklist: Recommended items to address in a clinical trial protocol and related documents.

**Additional file 2.** Model consent form – in Dutch.

**Additional file 3.** Intervention protocol template

## Data Availability

The datasets used and/or analyzed during the current study are available from the corresponding author on reasonable request.

## Abbreviations

ANOVA: Analysis of variance
CA: Corrected age
CVI: Cerebral visual impairment
METC: Medical ethical testing committee
PAI-CY: Participation and Activities Inventory – Children and Youth
RCT: Randomized controlled trial
RTF: Reaction time to fixation
VFA: Visual function assessment
VPD: Visual processing dysfunctions
