# Supplementary material for: Early intervention for children at risk of visual processing dysfunctions from 1 year of age: a randomized controlled trial protocol
Source: Trials. 2020 Jan 8;21:44. doi: 10.1186/s13063-019-3936-9 (PMC6950993; doi:10.1186/s13063-019-3936-9)
Supplement: Supplementary file 2 — Additional file 2. Model consent form – in Dutch. [file 13063_2019_3936_MOESM2_ESM.docx]

**Additional file 2 -** Model consent form – in Dutch


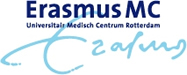


Toestemmingsformulier

**“Blik Vooruit”**

**Een visueel ontwikkelingsperspectief voor te vroeg geboren kinderen**

Ik ben gevraagd om toestemming te geven voor deelname van mijn kind aan dit medisch- wetenschappelijke onderzoek.

**Naam kind: Geboortedatum kind: ­____ /____ / ____**

Ik heb de informatiefolder (januari 2017- patiëntgroep) gelezen. Ook kon ik vragen stellen. Mijn vragen zijn voldoende beantwoord. Ik had genoeg tijd om te beslissen of ik wil dat mijn kind meedoet. Ik weet dat meedoen vrijwillig is. Ook weet ik dat ik op ieder moment kan beslissen dat mijn kind toch niet meedoet. Daarvoor hoef ik geen reden te geven.

Ik geef toestemming voor het informeren van de specialist die mijn kind behandelt dat mijn kind meedoet aan dit onderzoek. Ik geef toestemming voor het opvragen van informatie bij de huisarts/specialist die mijn kind behandelt over medische en (neuro)psychologische gegevens die van belang zijn voor het onderzoek.

Ik geef toestemming voor gebruik van de gegevens op de manier en voor de doelen die in de informatiebrief staan. Ik geef toestemming om de gegevens van mijn kind op de onderzoekslocatie nog 15 jaar na dit onderzoek te bewaren.

Ik geef **wel/ geen*** toestemming voor het maken van camera opnames van mijn kind tijdens de uitvoering van de visuele screening.

Ik geef **wel/geen*** toestemming om mijn kind na dit onderzoek opnieuw te benaderen voor een vervolgonderzoek of follow-up.

**Ik ga ermee akkoord dat mijn kind meedoet aan dit onderzoek.**

**Naam ouder/voogd**: Datum:­__ / __ / __**

**Handtekening:**

**Naam ouder/voogd**: Datum:__ / __ / __**

**Handtekening:**

***** Doorhalen wat niet van toepassing is.

****** Als het kind jonger dan 18 jaar is, ondertekenen de ouder die het gezag uitoefenen of de voogd dit formulier.

Graag onderstaande persoonsgegevens invullen, alleen als u toestemming heeft gegeven:

adres:

postcode:

woonplaats:

telefoonnummer overdag:

telefoonnummer ‘s avonds:

-----------------------------------------------------------------------------------------------------------------

Ik verklaar hierbij dat ik bovengenoemde persoon/personen volledig heb geïnformeerd over het genoemde onderzoek. Als er tijdens het onderzoek informatie bekend wordt die de toestemming van de ouder of voogd zou kunnen beïnvloeden, dan breng ik hem/haar daarvan tijdig op de hoogte.

Naam onderzoeker (of vertegenwoordiger):

Handtekening: Datum:___/___/___

-----------------------------------------------------------------------------------------------------------------
