# Supplementary material for: Early intervention for children at risk of visual processing dysfunctions from 1 year of age: a randomized controlled trial protocol
Source: Trials. 2020 Jan 8;21:44. doi: 10.1186/s13063-019-3936-9 (PMC6950993; doi:10.1186/s13063-019-3936-9)
Supplement: Supplementary file 3 — Additional file 3. Intervention protocol template [file 13063_2019_3936_MOESM3_ESM.docx]

**Additional file 3 -** Intervention protocol template

**Visual Intervention Protocol**

**For children aged ≥1 year**

(Authors: *Marlou Kooiker, Jenneke van Dijk, Yoni van der Linden)*

1. **PREPARATION & HABITUATION**

Week 1 to 4, once a week

Observation in the daily environment of the child, as an habituation period for therapist and family. Build trust and provide examples. Build proper connection and interaction with the child.

Optional: visiting daycare/ babysitter, but emphasis lies on behavior in the home environment. Incorporate social worker when there is a clear indication.

2-4 times observation in home environment and/or visit to daycare or babysitter (as often as needed), of which one observation with video evaluation (+ consent form needed)

Report the observations in a standardized and structural manner with the following instruments, depending on the visual level of the child:

Level B-C: - ZieZo observation method (Royal Dutch Visio)

Level D-E-F : - visual screening for young children (Visio Dordrecht)

All levels:

- Characteristics of cerebral visual impairment (CVI) and looking behavior, according to the template exploration report

- Oregon Skills list for children aged 0-2 years

Week 5: write Exploration report and make Intervention Plan (IP).

- Write the Exploration report according to the format for this study
  - Give focus and direction. State the goals and the instructions for parents.
  - Video evaluation (based on a video observation of the child) as basis for IP
  - Characteristics CVI and looking behavior
- Write the IP according to the format for this study

Week 6: Multidisciplinary meeting to discuss and approve final IP

1. **INTERVENTION**

Content of the program: activities and materials depend on the child and his/her circumstances, needs and preferences (see attachment A).

Standardized components:

Duration of intervention: approximately one year

Setting of intervention: at home and/or daycare

Number of sessions with therapist: once a week, later once every 2 weeks (minimum of 20 sessions)

Duration of sessions with therapist: 60 minutes + 30 minutes for report writing

Number of practice sessions for parents/caregivers: 15 minutes per day (3x5 minutes), minimum of 5 day a week

Registration of activities: therapists fill out a logfile per session, parents/caregivers fill out a logfile per week (reporting the practice sessions with their child).

**Global timeline**

*At the start of intervention the therapist will make a personalized time schedule in consultations with parents/caregivers*

Start of intervention in week 7: 1^st^ focus moment

Video feedback & evaluation with parents (result of multidisciplinary consultation week 5).

Week 7 to 14: habilitation once a week, 90 min per session.

Consisting of:

20 minutes Starting up, discussing the previous instructions (stepping card) for parents, evaluate what they did, how often, and how it went based on their logfile.

30-40 minutes Habilitation session

20 minutes Closing, consisting of evaluation and formulating a new instruction (stepping card; continuation or change). Writing session report.

Week 15: During habilitation session a video observation is made of the child. This can be consulted in a multidisciplinary meeting.

Week 16: 2nd Focus moment

Video feedback & evaluation with parents/caregivers

Fill out Oregon questionnaire (digital), and incorporate in session report.

Evaluation of the IP

From now on the habilitation will become less intensive: once every 2 weeks, or, in case habilitation is no longer indicated, once every 4 weeks.

From week 18: habilitation with therapist once every 2 weeks

Week 30: During habilitation session a video observation is made of the child. This can be consulted in a multidisciplinary meeting.

Week 32: 3^rd^ Focus moment

Video feedback & evaluation with parents/caregivers

Fill out Oregon questionnaire (digital), and incorporate in session report.

Evaluation of the IP

From week 32: continue until the child has reached the (corrected) age of 2 years

1. **WRAP UP**

At 2 years of (corrected) age:

- Repeat the observations:
  - Oregon skills list
  - CVI characteristics and looking behavior
  - ZieZo observation OR visual screening for young children (Royal Dutch Visio)
- Write Evaluation report (same format as exploration report; to map differences)
- Repetition of visual function assessment
- Determine whether habilitation ends, or whether there is an indication to continue (multidisciplinary consult)
